# Eyjafjallajökull and 9/11: The Impact of Large-Scale Disasters on Worldwide Mobility

**DOI:** 10.1371/journal.pone.0069829

**Published:** 2013-08-07

**Authors:** Olivia Woolley-Meza, Daniel Grady, Christian Thiemann, James P. Bagrow, Dirk Brockmann

**Affiliations:** 1 Engineering Sciences and Applied Mathematics, Northwestern University, Evanston, Illinois, United States of America; 2 Northwestern Institute on Complex Systems, Northwestern University, Evanston, Illinois, United States of America; 3 Max-Planck-Institute for Dynamics and Self-Organization, Göttingen, Germany; 4 Robert-Koch-Institute, Berlin, Germany; University of Namur, Belgium

## Abstract

Large-scale disasters that interfere with globalized socio-technical infrastructure, such as mobility and transportation networks, trigger high socio-economic costs. Although the origin of such events is often geographically confined, their impact reverberates through entire networks in ways that are poorly understood, difficult to assess, and even more difficult to predict. We investigate how the eruption of volcano Eyjafjallajökull, the September 11th terrorist attacks, and geographical disruptions in general interfere with worldwide mobility. To do this we track changes in effective distance in the worldwide air transportation network from the perspective of individual airports. We find that universal features exist across these events: airport susceptibilities to regional disruptions follow similar, strongly heterogeneous distributions that lack a scale. On the other hand, airports are more uniformly susceptible to attacks that target the most important hubs in the network, exhibiting a well-defined scale. The statistical behavior of susceptibility can be characterized by a single scaling exponent. Using scaling arguments that capture the interplay between individual airport characteristics and the structural properties of routes we can recover the exponent for all types of disruption. We find that the same mechanisms responsible for efficient passenger flow may also keep the system in a vulnerable state. Our approach can be applied to understand the impact of large, correlated disruptions in financial systems, ecosystems and other systems with a complex interaction structure between heterogeneous components.

## Introduction

The infrastructure that supports the flow of assets, energy, information and people at all scales often operates near maximum capacity, accentuating the vulnerability of socio-technical systems [1]. Even partial failure due to error, interference from environmental conditions, or malicious attacks [2]–[4] can lead to massive economic losses and social disruption. This infrastructure also has the unintended consequence of facilitating the rapid spread of emergent infectious diseases [5], [6] and intentional shutdown could become necessary to impede transmission [7], [8]. Modeling these systems as complex networks [9]–[12] we investigate methods for assessing their resilience to large-scale disruptions. Resilience of networks to both random failure and targeted attacks has been widely studied [2], [13]–[17]. However, there has been little investigation of system resilience to real disasters [18] which generically fall outside of these theoretical benchmarks. It remains unclear how natural events differ from idealized model systems, how they differ from one another and what features they share.

**Figure 1:**
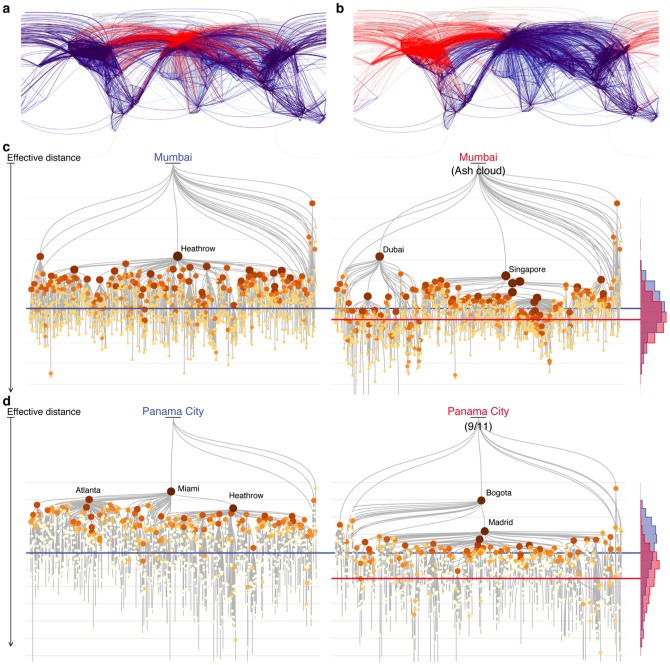
Quantifying the impact of disasters on the worldwide air transportation network (WAN). (**a**) The ash cloud led to the closure of more than 100 airports in the WAN. These closures interrupt all the routes shown in red, which account for 20.5% of total traffic. (**b**) The 9/11 terrorist attacks closed more than 200 airports, interrupting 37.7% of total traffic. (**c**) The shortest-path tree rooted at Mumbai (BOM) before (left) and after (right) the ash cloud closures. This tree captures the expected routes passengers take for trips originating at Mumbai (BOM). The ash cloud “pulls” the world away from Mumbai (BOM), increasing its effective distance to other airports as illustrated by the histogram of distances on the right. Blue and red correspond to distributions before and after the disruption, respectively. Horizontal lines mark the mean effective distance. An airport's size in the tree indicates how many other airports are reached from Mumbai (BOM) by traveling through that airport. We see that Heathrow is typically Mumbai's gateway to the world. When Heathrow is removed a new set of hubs become Mumbai's (BOM) primary access points. (**d**) From the perspective of Pananama City (PTY), a similar increase in effective distance is caused by the 9/11 closures, when Miami (MIA) is replaced by Bogota (BOG) as the gateway to the world.

Here, we investigate how two large-scale disasters impact global mobility through disruptions of the worldwide air transportation network (WAN) [19]–[21]: the 2010 eruption of the Icelandic volcano Eyjafjallajökull and the terrorist attacks of September 11th, 2001 (illustrated in Figs. 1a and 1b respectively). Although these events only closed airports within bounded geographical regions, their effects echoed throughout the global traffic network and generated large economic losses everywhere. The International Air Transport Association estimated that airlines alone lost more than 1.7 billion USD in the six days following Eyjafjallajökull's eruption, and approximately 10 million passengers were affected [22]. However, theoretical percolation approaches that focus on the topological integrity of a network and which are frequently used to understand network resilience [13], [14] fail to capture this marked socio-economic impact because neither event compromised the global integrity of the WAN (see Text S1 Sec. S4.1). In fact, the effects of these events vary widely between airports and regions; while some areas function close to normal, others may be all but obliterated.

To successfully capture this impact and explain its variability, we employ the concept of node-specific perspectives of the network [23] and of *effective distance*. The essence of our approach is illustrated in Fig. 1c, d which shows the most efficient routes from the perspective of two different reference airports, Mumbai (BOM) and Panama City (PTY). Most traffic is routed through central gateway airports, such as Heathrow (LHR) or Miami (MIA). When these gateway airports close due to a disruption such as the volcanic ash cloud or 9/11, efficient routes change and a new set of airports become Mumbai's (or Panama's) primary access points. Therefore the overall effective distance from each one of these two airports to the rest of the world increases.

Using this view we capture at fine granularity how the impact of disruptions varies across different locations. We find that the susceptibility of an airport to a specific disruption is inversely related to the number of connections at the airport since these provide routing flexibility. Therefore, susceptibilities to real events, geographical events and random events follow similar, highly heterogeneous statistical distributions. However, because global routing relies heavily on a set of core airports, susceptibilities to attacks that target only this core are more uniform. Our approach captures and enables us to explain the degree of heterogeneity in a system's response to disruptions, a previously overlooked yet key factor in the vulnerability of the WAN and other infrastructural networks that support heterogeneous flows.

## Results

The worldwide air transportation network (WAN) is the global network of airports that are connected through direct flights. Approximately 3.1 billion passengers travel through this network per year. A connection 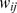 between airports 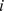 and 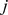 represents the total number of passengers traveling between these airports in a year (see Text S1 Sec. S1 for more detailed information on the WAN dataset). The *flux* 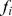 of airport 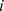 is the total traffic traveling through it: 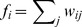, while airport *degree* 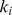 is the number of other airports to which 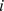 has direct flights: 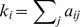, where 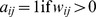 and 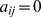 otherwise.

We model the impact of Eyjafjallajökull and 9/11 on the WAN by removing the same set of airports that were closed in response to these events (see Text S1 Sec. S2.1) together with the non-stop flights to and from these airports. Our method captures the dynamic re-routing of passengers at functional airports to avoid obstructed multi-stop connections through airports that close. At their peak, on April 16th 2010, the closures due to Eyjafjallajökull's ash cloud interrupted 20.5% of the total traffic and closed 10.5% of all airports. The closure of American and Canadian airspace as a response to the 9/11 attacks was even more severe, removing 37.7% of air traffic and 19.6% of all airports. In light of these disruptions, how well can the remaining airports sustain global connectivity and how does susceptibility vary across different airports and regions?

**Figure 2:**
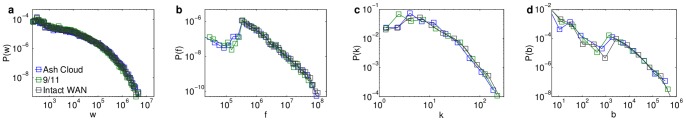
Network properties before and after natural disruptions. **(a–d)** Relative frequencies of standard network measures: link weight 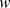, node flux 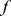, degree 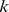, and betweenness 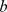. The functional form of the probability distributions are largely unaffected by the events under consideration.

Common network diagnostics are the distributions of flux 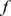, degree 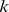 and more sophisticated centrality measures such as betweenness 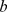 [9], the number of most-efficient paths between all other node pairs that traverse a node. In a large variety of networks, in particular mobility and transportation networks including the WAN, centrality measures are broadly distributed reflecting the strong structural heterogeneity of the network [24], [25]. A plausible approach to assess the impact of a natural, large-scale disruption is to measure systematic changes in the distributional form of these standard centrality measures. However, we find that the functional form of degree, flux, and betweenness distributions is surprisingly robust to these disruptions as illustrated in Fig. 2. Thus, changes in said distributions are not suitable for assessing the impact of these disasters.

On the other hand, from the perspective of individual airports, changes in the efficiency of network connectivity are apparent as we have seen in Figs. 1c, d. In addition to taking a node's perspective, the key element of our approach is to measure the increase in *effective distance* [26] due to this decay in connectivity. Effective distance is based on the intuitive notion that strongly connected nodes are effectively “closer” than those that are connected by a weak link. More specifically, we first define the effective length 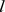 of a path that starts at node 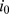 and ends at 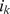, passing through intermediate nodes 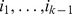:
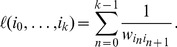
(1)


Given the set 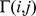 of paths that connect nodes 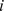 and 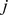 we define the effective distance 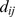 as the length of the *shortest* path between them:
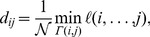
(2)where the normalization is arbitrary and chosen such that the global mean effective distance in the intact network is 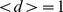. Note that two airports that have a weak direct connection may nonetheless have a small effective distance between them if they have a strong multi-stop connection.

For each pair of airports we denote the change in effective distance as 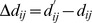, where prime indicates quantity after a disruption. Note that the effective distance between two airports is only defined when they are connected. Thus, we will only take into account changes in effective distance between a pair of airports 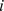 and 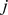 if they remain connected after a disruption.

In order to understand the nature and effects of natural disruptions we compare them to synthetic control scenarios of comparable overall magnitude, controls A and B corresponding to the ash cloud and 9/11, respectively. We classify controls as **random**: airports are equally likely to be removed, **targeted**: airports are chosen in order of decreasing centrality according to degree, flux, and betweenness, and **geographic**: airports are closed based on spatial distance from a given epicenter.

Interestingly, we find that although the 9/11 disruption was more severe in terms of overall traffic reduction, the ash cloud event pulled the world farther apart. For instance, the mean shift in effective distance over all pairs of airports 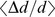 is approximately 0.557 following the ash cloud vs. 0.0923 following 9/11. To quantify the relative size of their impact, we compare the shift 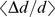 in the two real events to randomly selected synthetic geographic disruptions that remove comparable amounts of traffic. We find that the ash cloud disruptions are exceptionally strong (only 14% of synthetic disruptions exhibit a greater shift) while the 9/11 attacks are slightly weaker than expected (up to 60% of synthetic disruptions exhibit a larger shift). Furthermore, large-scale geographically clustered disruptions exhibit large global shifts only when they shut down Western Europe. Although this is partly due to the density of high-betweenness airports in this region, betweenness alone cannot explain the exceptional sensitivity to the closure of European hubs (see Text S1 Sec. S4.3 for more details).

**Figure 3:**
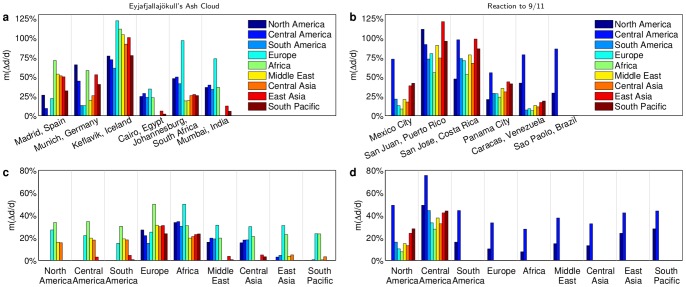
The geographical distribution of impact in real-world disruptions. **(a, b)** The panels depict percent airport-to-region distance shift, i.e. the median change in effective distance from a given airport to all airports in a specific region. An airport's shift profile reveals fine-grained information about the regional structure of its connections. For instance, shifts are generally larger for airports geographically close to the disruption epicenter, indicating strong within-region connections. We also see more idiosyncratic features, for instance, Madrid is close to the ash cloud epicenter but exhibits a relatively small shift from Latin America because it is strongly connected to it. **(c, d)** Panels depict percent inter-regional distance shift, i.e. the median change in effective distance from all airports in a specific region to all those in a second region. When the ash cloud shuts down many European airports, Europe predictably experiences a strong distance shift throughout. More interestingly, Africa experiences a comparable shift and all regions shift away from geographically distant regions. Meanwhile, shifts due to the 9/11 closures are confined to North and Central America, evidence that European hubs are more crucial to inter-regional connectivity.

Global shifts contain no information about how the susceptibility to a given disruption varies across different airports and regions. To capture this aspect of network resilience we instead quantify impact from the perspective of individual airports. In Figs. 1c, d we can clearly see that the change of distance along shortest paths from Mumbai (BOM) and Panama (PTY) varies from one airport to another. In geographical disruptions we expect that because of the underlying spatial and political constraints [19], [27] variation will partly follow geopolitical boundaries. This is in fact what we see in Fig. 3a, b which shows the distance shift from individual airports to different geopolitical regions. Generalizing this idea to capture the distance shift between pairs of geopolitical regions reveals that the impact of the ash cloud closures had a greater geographical scope than the 9/11 closures (see Fig. 3c, d).

Despite these fine-grained differences between disruptions, a key question is whether impact magnitude follows general and possibly universal distributions due to the underlying network structure. The overall impact at an airport 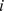 is given by the change in its typical effective distance to all other airports. We define an airport's *susceptibility* by
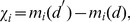
(3)where 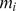 denotes the median of the distribution of 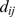 for fixed 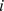, and the prime denotes the situation after the disruption. Again, for each 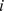 we only consider the set of airports to which it is connected before and after the specific disruption.

**Figure 4:**
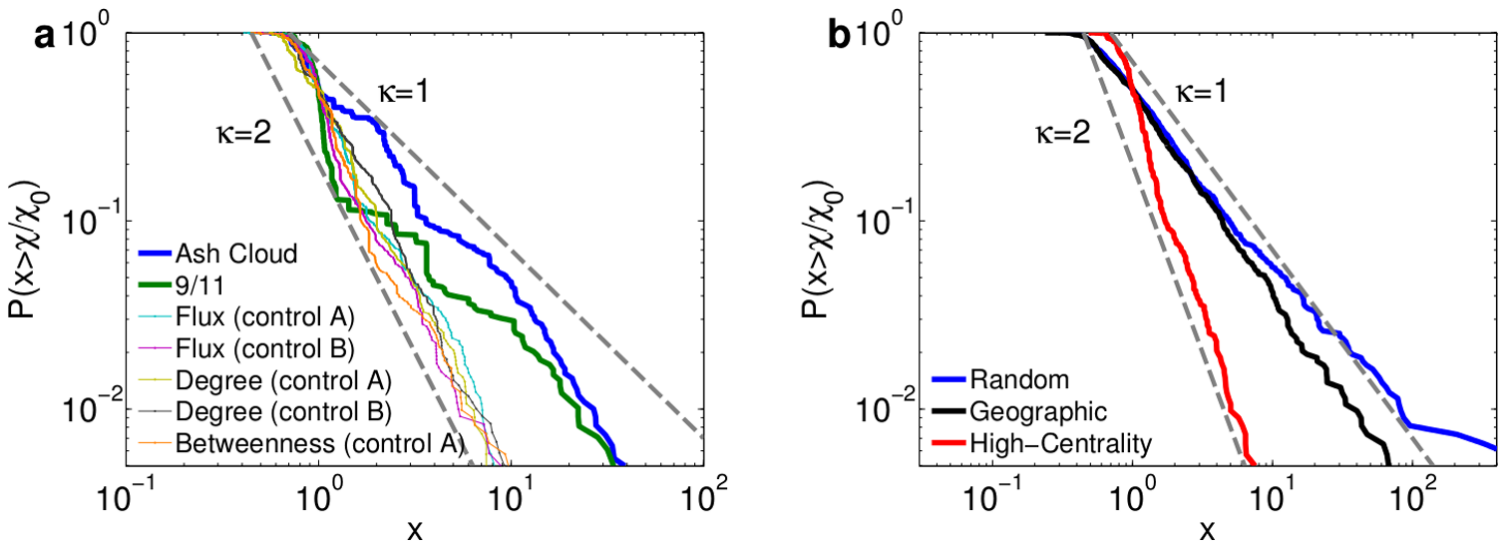
Distributions of airport susceptibility. (**a**) The cumulative probability distributions 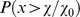 capture the strong impact heterogeneity across airports in all disruptions. Susceptibilities to real scenarios are more broadly distributed than in attacks targeting central airports. Dashed lines mark power laws 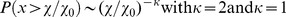. (**b**) Same as panel (a) for the ensembles of geographical and random disruptions and attacks that target high-centrality airports (for details see Text S1 Sec. S5). The ensembles of random an geographical disruptions follow a similar scaling to the real-world disruptions (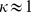) while the targeted attack ensembles scale like individual high-centrality disruptions (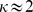).

Interestingly, we find that in natural disruptions as well as the synthetic control scenarios, the distribution of airport susceptibilities 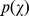 has a broad tail that can be described by
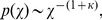
(4)as shown in Fig. 4a, b. (For statistical tests supporting the plausibility of this power-law distributional form see Text S1 Sec. S5.3.) Different types of disruption scenarios exhibit different tail exponents 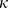: all high-centrality targeted attacks exhibit a similar scaling exponent 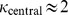 yet the distribution of susceptibility to the natural events is broader, with 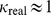. This means that the latter generate more variable impact. Impact distributions of synthetic, geographically confined disruptions possess exponents similar to the natural events (Fig. 4b), as we would expect given the geographical coherence of natural disruptions. Note that the scaling exponent in random disruptions is also comparable to natural and geographical events (Fig. 4b). Thus, disruptions with very different geographical structures can have similar distributions of susceptibility, suggesting that this behavior cannot be explained by geographic characteristics alone.

What are the network properties that can account for the observed behavior under different types of disruptions? Generically, in networks with broad weight and degree distributions, only a small subset of links make up the essential connectivity structure [23], [28]. In hierarchical networks [29] such as the WAN, these connections are generally those that channel efficient navigation from the periphery of the network to the core, along increasingly central airports [20]. This essential backbone is of the order of the number of nodes in the network and it is tree-like away from the core [23]. At an individual airport this will lead to a *heterogeneous* distribution of most-efficient paths across its links. This structural heterogeneity governs the behavior of impact. To understand how, we will identify the subset of essential connections using the high-salience skeleton [23], a method designed specifically for this purpose.

Consider an airport that remains open following a disruption. If this airport loses a link in the high-salience skeleton due to a disruption that closes the destination of that link, we say that this airport experiences *direct* impact. Impact due to changes further removed from the airport is referred to as *indirect*. Both types of impact can contribute to an airport's susceptibility: 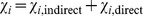.

**Figure 5:**
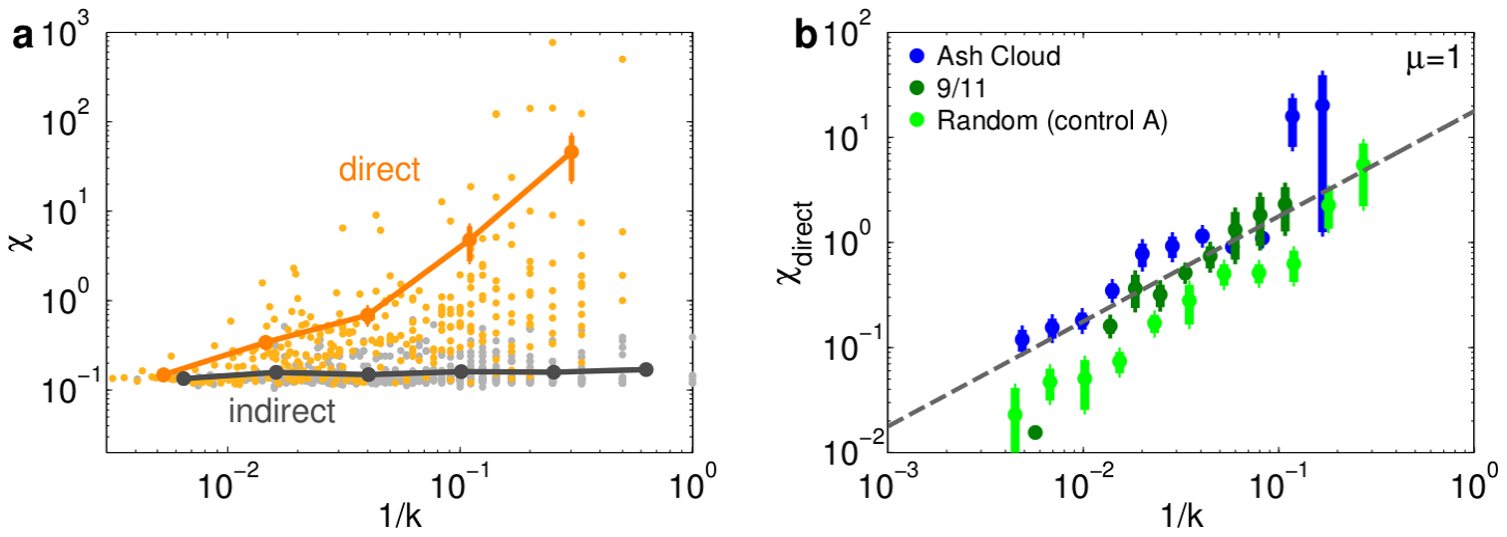
The degree distribution drives direct susceptibility. (Error bars indicate 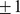 se.) (**a**) Susceptibility 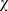 as a function of airport inverse degree 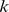 for directly and indirectly affected airports in a random disruption. We observe a clear difference in behavior between airports that lose a crucial link (*direct* impact) and those which experience only *indirect* impact. Direct impact increases as 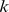 decreases, while indirect impact is approximately constant. Solid lines denote the averaged trend. (**b**) Direct susceptibility 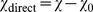 of directly affected airports as a function of inverse degree reveals the scaling 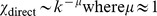.

In disruptions that remove a non-negligible number of airports, such as those we consider, all airports experience indirect impact. We expect that indirect susceptibilities are approximately uniform since these changes typically occur far away, affecting a small number of routes. This implies that airports that do not experience direct impact are approximately uniformly susceptible 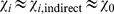. We confirm this in Figure 5a. Since susceptibility is a change of effective distance, 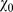 can be interpreted as the typical distance change from the network core to all other airports (see Text S1 Sec. S7 for more explicit calculations and discussion).

The situation is more subtle for airports that are directly affected. Following a disruption these airports rely on their remaining direct connections to mitigate the loss of crucial direct connections. Assuming that an airport's rerouting flexibility, i.e. the number of existing links across which rerouted traffic can be distributed, increases with degree, we expect that susceptibility decreases with degree. This reasoning is confirmed in Fig. 5b, which shows that
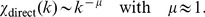
(5)


Based on this scaling relation and the degree distribution we expect 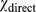 to be distributed according to
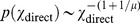
(6)(this simple scaling argument is detailed in Text S1 Sec. S8). Given that 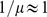, this is consistent with the empirical finding corresponding to natural, geographical and random disruptions shown in Fig. 4, and thus the rerouting flexibility captured by airport degree suffices to explain the distribution of susceptibilities to these events.

However, the above scaling argument fails to explain the distribution of impact following targeted attacks. The argument rests on the assumption that the direct impact dominates. Targeted attacks cripple the core of the network, shutting down multiple connections that are critical for long-range travel, in this way indirectly impairing efficient routes to and from most airports. Thus, contrary to the assumption above, indirect susceptibility dominates, yielding a more homogeneous distribution of 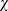 (i.e. a larger tail exponent 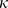).

**Figure 6:**
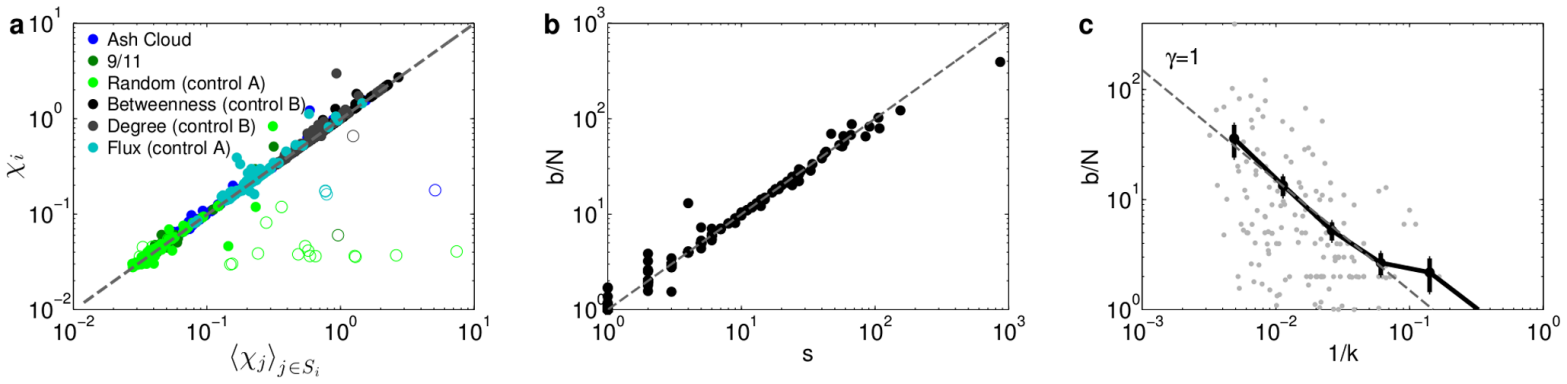
The distribution of indirect susceptibility is governed by the dependence structure. (Error bars indicate 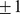 se.) (**a**) The susceptibility 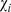 of an airport 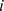 is approximately equal to the average susceptibility of its dependents 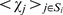 when the dependents are not directly impacted (solid circles). Consistent with our model of how impact spreads, when dependents are directly impacted (empty circles) their direct impact dominates, most notably in random disruptions. (**b**) The number of dependents 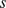 of an airport is approximately equal to its betweenness centrality 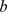 divided by the number of nodes 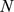. (**c**) Thus, both 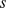 and 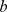 scale with degree 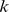 according to 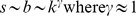.

To characterize this increased homogeneity in centralized attacks we need to determine how the impact at an airport affects others indirectly. Let the dependents of an airport 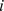 be the airports that rely on 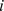 to access most of the network (at least half of the total 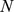 airports). If 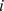 experiences direct impact, many routes from any one of its dependents will be disrupted. These dependents can in principle find another gateway to replace 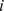, but even after damage 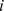 will typically continue to provide the most efficient connection. Thus we expect that rerouting flexibility at 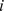 will indirectly affect travel from its dependents and in the absence of other effects they will exhibit approximately the same susceptibility as 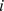. We verify in Fig. 6a that the dependents of an airport do on average exhibit the same susceptibility as their gateway.

Interestingly, we find that the number of dependents is approximately the per-airport betweenness centrality 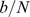 (see Fig. 6b). This relationship holds because the WAN has a tree-like backbone of efficient connections (if all of a dependent's most efficient routes go through the same gateway this relationship is exact; see [30] and Text S1 Sec. S9.1). Both the number of dependents and betweenness increase with airport degree 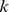 according to
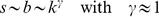
(7)as shown in Fig. 6c. Using this scaling, the probability that a randomly chosen airport will be a dependent of an airport of degree 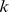 is proportional to 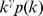. Each one of these dependents exhibits approximately the same susceptibility (indirectly) as this gateway does directly, and a simple calculation (detailed in Text S1 Sec. S9.2) yields the distribution of indirect susceptibilities, which dominate the full susceptibility distribution:
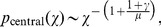
(8)where 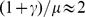. This result is consistent with the observed tail exponent 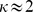 in targeted disruptions (see Fig. 4). Intuitively, Eq. (8) tells us that because betweenness centrality 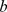 increases with the degree 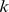 of a node (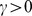), the network's dependence structure makes large variations in susceptibility less likely when the core of the network is targeted, narrowing the distribution of susceptibilities.

Different infrastructural networks can in principle exhibit different betweenness-degree centrality scalings [31]. The specific scaling relation in the WAN, captured by Eq. (7), together with Eq. (8) means that the system's response to geographical and random disruptions is qualitatively different from the response to disruptions that target central airports. To see how, consider that following the distributional form in Eq. (4), the variance scales (to leading order) with the sample size 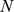 according to 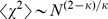 [32]. Thus, when 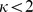 the variance diverges as 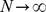. A generic exponent 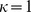 for natural disruptions (as well as geographical and random) implies they lack a scale. On the other hand, for targeted attacks in a network where the number of dependents increases sufficiently quickly with node degree to satisfy 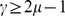, the variance converges. In the WAN 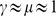 and thus targeted disruptions have a finite scale.

## Discussion

We have characterized the effects of disruptions to the WAN on worldwide mobility using simple scaling arguments based on basic network properties. This reveals not only how the system responds to large-scale disruptions, but what key properties of the system are necessary to predict and explain this response. Furthermore, this analysis suggests some avenues for improving the resilience of global mobility to these disruptions. The WAN does not depend as heavily on physical infrastructure as other networks (e.g. the power grid). Thus, potential increases to the resilience and efficiency of the mobility it sustains could be implemented more rapidly through changes to airline scheduling.

We find that the Eyjafjallajökull event had an anomalously strong impact on worldwide mobility because the Western European airports forced to close provide irreplaceable connectivity between distant regions. This dangerous vulnerability can be minimized by introducing more high-traffic, inter-regional connections between non-European airports. Many of these connections are underutilized or nonexistent despite the lack of clear physical or logistical constraints.

We also discover that the structure of the WAN generates a tradeoff between the magnitude of local effects and their global reach. Peripheral airports are on average highly susceptible to direct impact because they rely strongly on a small number of links and have limited routing flexibility, but their indirect effect on other airports is negligible. Conversely, core airports are less susceptible to direct impact given their high connection redundancy, but many others are indirectly affected through them. Thus the network core is more resilient than the periphery. Even when events heavily target and damage the core, there is a well-defined impact scale that can be used to anticipate the consequences of these events. On the other hand, the impact of geographical and random events does not have a scale and is therefore harder to predict. These findings suggest that to optimize the resilience of worldwide mobility it may be necessary to look beyond the hub airports and reduce the susceptibility of peripheral airports, for example by distributing their traffic over a wider range of connections, spanning locations as geographically widespread as is economically and logistically feasible. Although this may compromise the efficiency of operations under normal conditions it may nonetheless pay off if geographical disruptions occur frequently. Future work should investigate how to restructure connectivity or build excess capacity for contingency re-routing in a way that balances efficiency under normal operations with the risk posed by these disruptions. Another important future avenue is to investigate network designs that do not exhibit a tradeoff between efficiency and resilience.

Beyond mobility and transportation networks, the framework of effective distance and a node's perspective can be used to assess and optimize the resilience of other systems with heterogeneous flows. For example, the interplay of direct and indirect impact could help understand how diversification by different players in a network of financial flows affects the complex tradeoff between systemic and individual risk [33].

## Supporting Information

Text S1 **Supplementary text, figures and tables.**
(PDF)
