# Supplementary material for: Eyjafjallajökull and 9/11: The Impact of Large-Scale Disasters on Worldwide Mobility
Source: PLoS One. 2013 Aug 7;8(8):e69829. doi: 10.1371/journal.pone.0069829 (PMC3737197; doi:10.1371/journal.pone.0069829)
Supplement: Text S1 — Supplementary text, figures and tables. (PDF) [file pone.0069829.s001.pdf]

Supplementary Information

Eyjafjallajökull and 9/11: the impact of large-scale disasters on worldwide mobility
by Olivia Woolley-Meza, Daniel Grady, Christian Thiemann, James P. Bagrow and Dirk Brockmann

Table of Contents

S1 Dataset **2**
S1.1 Description 2
S1.2 The symmetry of travel 2
S1.3 Yearly variation in data and the robustness of results across different years 2
S1.4 The reduced network 3

S2 Representing airport closures **3**
S2.1 Real-world events 3
S2.2 High-centrality attacks 4

S3 Effective distance **4**

S4 Global measures of network resilience **6**
S4.1 Topological percolation 6
S4.2 Inflation of diameter 6
S4.3 Europe as a global bridge 7

S5 Characterizing the distribution of airport susceptibilities **9**
S5.1 From single events to impact ensembles 9
S5.2 The differences between geographical and random disruptions 9
S5.3 Fitting the scaling exponent of the susceptibility distribution 10

S6 Hierarchical structure of shortest paths **10**
S6.1 Modeling the structure of efficient routes 10
S6.2 The center of the network 12

S7 Interpreting the decomposition of susceptibility and estimating background impact **14**

S8 Scaling of susceptibility distributions when direct impact dominates **15**
S8.1 Susceptibility and degree 15
S8.2 Directly impacted airports according to disruption type 15
S8.3 Change of variables 16

S9 Scaling of susceptibility distributions when indirect impact dominates **17**
S9.1 Measuring the relationship between degree and number of dependents 17
S9.2 Indirect impact narrows the distribution of susceptibility 19

References **19**

List of Figures

S1 Comparing the properties of the WAN in 2004 to those in 2006 3
S2 Diameter of the WAN as a fraction of its original diameter as the smallest airports are removed . . . 4
S3 Effective distances capture the structure of both non-stop and multi-stop trips 5

S4 Topological percolation . . . 6
S5 Inflation of effective distances . . . 7
S6 The global impact of geographical atacks . . . 8
S7 Impact averaged over random and geographical ensembles . . . 9
S8 Effective distances and hierarchy in the WAN . . . 11
S9 Heathrow's central role . . . 12
S10 The core of the network . . . 13
S11 Variation in direct disruptions with airport degree . . . 15
S12 Degree distribution of directly impacted airports . . . 16
S13 Direct impact distribution through degree inversion . . . 17
S14 Scaling relationships for the number of dependents . . . 18

List of Tables

S1 Regional breakdown of high-centrality disruptions . . . 5
S2 Betweenness centrality of regions . . . 8
S3 Fitting a power-law distribution to the susceptibility distributions . . . 10

S1 Dataset

S1.1 Description

We use a dataset provided by OAG (Official Airline Guide) Ltd. (http://www.oag.com), containing $N = 4,092$ airports and the number of seats on scheduled commercial flights between pairs of airports during the years 2004–2006. The number of seats on scheduled commercial flights from airport i to j is given by $\tilde{w}_{ij}$, which we take to be proportional to the number of passengers traveling.

S1.2 The symmetry of travel

We define w_{ij} as the total number of passengers that travel between i and j. We carry out our analysis on the undirected network with weight matrix with elements w_{ij} defined by $W_{ij} = \tilde{W}_{ij} + \tilde{W}_{ji}$. Because traffic is strongly symmetric for all airports i and j, this simplification retains most information on the structure of air travel. Concretely, if we let X and Y be the vectors made up by the elements of the ordered row vectors of W_{ij} and W_{ij}^T respectively, the Pearson correlation [1] of X and Y is 0.999, which shows that they are strongly linearly related according to $Y = X$. The normalized root mean squared deviation (NRMSD) [1] is 0.0282%, indicating that variation from equality is small overall.

S1.3 Yearly variation in data and the robustness of results across different years

As discussed above, our analysis is based on data that records traffic flows in the period 2004-2006. This time-period does not overlap with the time of the real world events we investigate. However, we find that the WAN does not change at a rate that would significantly affect our results. Although traffic flows are steadily increasing in time, according to an OAG report [2] total capacity increased at a yearly rate of only 2.6% from 2001 to 2010. Furthermore, our results will not change unless the structure of the network changes or the relative capacity along different routes is modified **disproportionately**. Using the data available to us, we compare simple properties of the WAN in 2004 to those of the WAN in 2006 (see Fig. S1). We can see that although traffic and connections have increased, in general they do so proportionally – that is, there

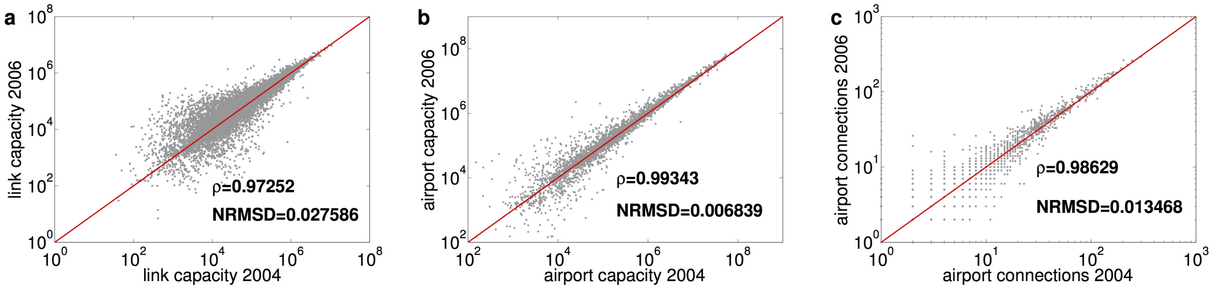

Figure S1: Comparing the properties of the WAN in 2004 to those in 2006. The correlation of network properties during this time frame is so strong that it is highly unlikely that the network evolution is fast enough to invalidate our results during either 2001 or 2010. We observe some variation from the expectation, as the smaller connections suddenly increase their weight by an order of magnitude. However, the average deviations are small as quantified by the normalized root-mean-squared deviations (NRMSD).

is no evidence for bias. We also see that the correlation coefficients of the individual link strength, and the number of connections and flights from each airport in 2004 and 2006 are very high, and that the NRMSD is small. Furthermore, we note that the change in the network is not in the same direction over time for all airports – while traffic increases in some parts of the network, in others it decreases. We also find that the impact behavior using the 2004 or the 2006 data is qualitatively the same as that found using the period average. This is good evidence that any changes to the WAN over a small number of years are not enough to change the results of our statistical analysis.

S1.4 The reduced network

Most airports in this dataset serve very few passengers; though less important, these airports affect quantities averaged over the network due to their large number. To reduce the network in a systematic way, we investigate how the weighted diameter of the WAN changes as the smallest airports are removed (Fig. S2). We track the diameter of the network [3] using the maximum and mean pairwise effective distances d_{ij}. Starting with the full network and removing airports in order of increasing airport traffic, we find that the diameter exhibits a marked discontinuous decrease when the smallest airports accounting for 5% of the total traffic have been removed. Thus, to minimize bias in average path-length statistics we reduce the network to the 1,227 airports with greatest traffic through them, accounting for 95% of total traffic. It is important to note that this network reduction does not qualitatively change results on the behavior of impact distribution at the airport level: exponents are similar in both networks and attacks targeting the center of the network are consistently narrower than the other classes of disruptions.

S2 Representing airport closures

S2.1 Real-world events

We remove the following countries and the airports from the network to reflect the real-world scenarios [4] (in parentheses the number of airports removed from the 95%-flux network):

- *Ash Cloud:* Belgium (2), Denmark (5), Estonia (1), Finland (7), France (partly; 8), Germany (partly; 21), Ireland (6), Latvia (1), Luxemburg (1), Netherlands (4), Norway (14), Poland (6), Sweden (15), United Kingdom (36); total: 127 (+2)

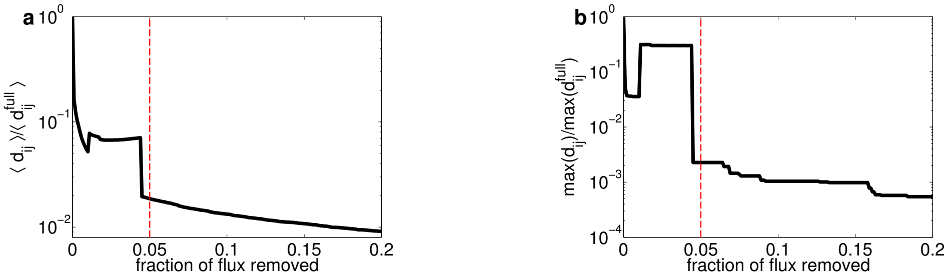

Figure S2: Diameter of the WAN as a fraction of its original diameter as the smallest airports are removed. (**a**) When the least busy airports accounting for 5% of the flux (red dashed lines) have been removed, the network diameter (as given by the mean effective distance), is steeply reduced. It is important to choose the network reduction carefully since the presence of these very small airports can bias measurements of the average resilience of the network. Thus, we reduce the network by removing these 5% smallest airports. (**b**) We can see the same steep decline in global distances when we measure the diameter of the network using the maximum effective distance.

This list reflects the airspaces closed before the afternoon of April 16, 2010. At this time, no flights following visual flight rules (VFR) were performed. Some airports allowed a small number of flights which we do not account for, e.g. from Dublin (Ireland) to the Americas, or selected low-altitude domestic flights. Only the northern parts of France and Germany were affected and we approximated this division by closing all airports north of the line running through Munich (Germany) and Krakow (Poland), with Munich being open, but Krakow closed. The two French airports Carcassonne (CCF) and Nimes (FNI) were not explicitly closed but disconnected from the network, thus a total of 129 airports is missing in the ash cloud scenario.

- *9/11:* United States (212), Canada (29); total: 241

S2.2 High-centrality attacks

For high centrality attacks we use degree, flux and betweenness. Although centrality measures are correlated, we can see that the airports removed by different targeted attacks are not the same. We consider all of these measures under two cases: traffic removal equivalent to the ash cloud closures (**control A**) and traffic removal equivalent to the 9/11 closures (**control B**). This makes six different attack scenarios. In total, 26 different airports are removed by at least one of these six attacks. All of the high-centrality attacks that we consider remove Heathrow(LHR), Charles De Gaulle (CDG), Frankfurt (FRA) and Atlanta (ATL). On the other hand, most airports do not appear in many attacks – for instance, 14 airports are removed by only one of the high-centrality attacks. Attacks which remove high flux airports target more airports in North America, while those that remove based on degree and betweenness target more in Europe (see Table S1). In Sec. S4.3 we discuss further the variation of different centrality properties with geography and what role this plays on the impact of geographical attacks.

S3 Effective distance

Effective distance is the distance along shortest-paths between every pair of airports, where the shortest-paths are not necessarily non-stop flights. In fact, we find that in the dataset that we use for our analysis 99.64% of all shortest-path routes are multi-stop. It is important to note that the effective distance between two airports is not necessarily the same as the direct shortest topological distance. Consider the main airports of Nashville

	Flux (control A)	Flux (control B)	Degree (control A)	Degree (control B)
North America	0.50	0.50	0.36	0.43
Europe	0.38	0.25	0.64	0.54
Other	0.13	0.25	0	0.036

Table S1: Regional breakdown of high-centrality disruptions. Fraction of the removed airports that are within each region for high-flux and high-degree attacks. Control A denotes attacks that remove the same amount of traffic as the ash cloud closures and control B denotes the 9/11 equivalents. Note that the majority of removed airports are in Europe or North America for all disruptions, and that the high-flux disruptions remove mainly North American Airports while the high-degree focus on European airports.

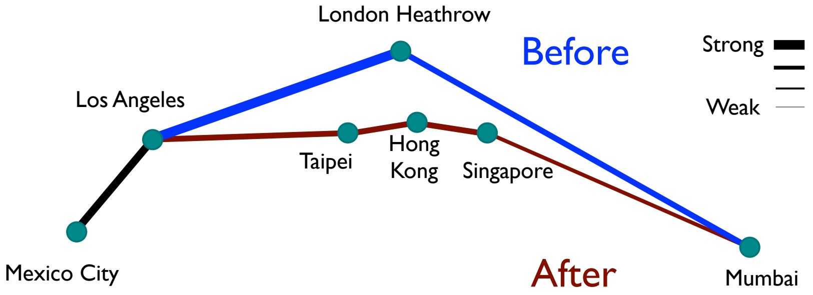

Figure S3: Effective distances capture the structure of both non-stop and multi-stop trips. Although there are many routes between any pair of airports, some are much more time and resource efficient than others – the routes that minimize total effective distance are a feasible approximation of these optimum routes. For example, in the intact network a traveller wishing to fly from Mexico City (MEX) to Mumbai (BOM) will likely travel through the high traffic routes to Los Angeles (LAX) and Heathrow (LHR). After the ash cloud disruption and consequent closure of Heathrow, a passenger will have to change their multi-stop route to a path less-travelled and more costly. This best remaining path is identified through the smallest updated effective distance, and in this case is the flight through a sequence of East Asian airports instead of flying through Heathrow. Thus, we can see that changes in effective distance **do** account for rerouting of multi-stop trips.

(BNA), Atlanta (ATL), and Los Angeles (LAX). Although there are many direct flights from Nashville to Los Angeles ($W_{\text{BNA,LAX}} = 198,380$ passengers), the path from Nashville to Atlanta to Los Angeles carries so much more traffic ($W_{\text{BNA,ATL}} = 392,280$ passengers and $W_{\text{ATL,LAX}} = 1,041,405$ passengers) that we have

$$\frac{1}{W_{\text{BNA,ATL}}} + \frac{1}{W_{\text{ATL,LAX}}} < \frac{1}{W_{\text{BNA,LAX}}}$$

Thus, the effective distance approximately reproduces the routing behavior that passengers experience when they are sent through hubs that may not be intuitively along their geographic travel path.

When an airport is removed together with all the non-stop flights to and form this airport, **changes** to the shortest routes (and therefore the effective distance) occur for all multi-stop trips that use these segments at any step of their path. For example, in the intact network passengers typically travel from Mexico (MEX) to Mumbai (BOM) along the shortest route MEX-LAX-LHR-BOM but due to the ash cloud closures (which close down LHR) they would typically now travel through Taipei (TPE), Hong Kong (HKG) and Singapore (SIN) on the route MEX-LAX-TPE-HKG-SIN-BOM (see Fig S3), which is a much more time consuming and costly route than the original one, but is the shortest path remaining.

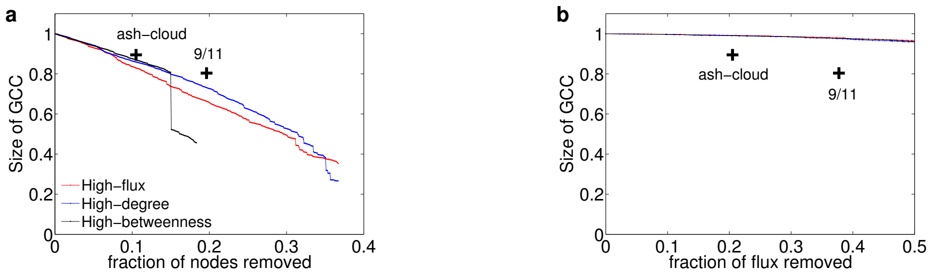

Figure S4: Topological percolation. (**a**) The size of the giant connected component (GCC) decreases as nodes in the network are removed in order of decreasing centrality according to flux, degree and betweenness. Initially, the size of the GCC decreases linearly with the fraction of nodes removed, indicating that most of the remaining airports are in the GCC. After a significant fraction of airports has been removed – 15% of airports removed in order of decreasing betweenness centrality or 35% in order of decreasing flux and degree – the size of the GCC decreases more rapidly as the network fragments. The crosses represent the fraction of airports removed and the size of the GCC after the real world events. In both events the size of the GCC is reduced only by the number of airports closed. Thus, the size of the GCC does not capture the severity of these disruptions. (**b**) Tracking the size of the GCC in terms of fraction of *flux* removed emphasizes the size of the reduction needed to fragment the network through high-centrality attacks. Although most of the flux is concentrated in a few nodes, weaker connections can hold the network together.

S4 Global measures of network resilience

S4.1 Topological percolation

A widely used indicator of network integrity is the size of the largest connected component. This maps resilience to a percolation problem, where the aim is to determine the number of nodes (or links) that must be removed through a specific attack to reach a threshold at which the network disintegrates into small components. From this perspective the WAN is very resilient (Fig. S4). The real-world attacks and even their high-centrality equivalents do not fragment the network. This is especially noticeable if we track the size of the component relative to the flux removed (Fig. S4b). Thus, the size of the giant component is not a useful measure for the resilience of the WAN to these types of events.

S4.2 Inflation of diameter

The median global shift in distances $m(\Delta d/d)$ shows a clear increase after only a small fraction of the flux is removed under high centrality attacks (Fig. S5a). According to this measure, when two attacks remove the same amount of flux, the most damaging are the ones that remove more betweenness (the high-betweenness attacks). However, if we measure impact through the mean global distance shift $\langle \Delta d/d \rangle$ instead, the random attacks appear more damaging than the central (Fig. S5b). The distribution of $\Delta d/d$ reveals why the two measures behave differently. As airports are removed in order of centrality (Fig. S5c illustrates the case of flux centrality) the shortest path distances of most airports increase slowly but a small number experience extreme distancing. This is captured by the emergence of additional peaks in Fig. S5c in the distancing distribution at 25% and 44% flux reduction. The mean $\langle \Delta d/d \rangle$ reacts sensitively to these single-airport effects, while the median $m(\Delta d/d)$ is more stable. One of the advantages of investigating impact at the level of airports is that it contains explicit information about impact heterogeneity, at a granularity that can be easily understood.

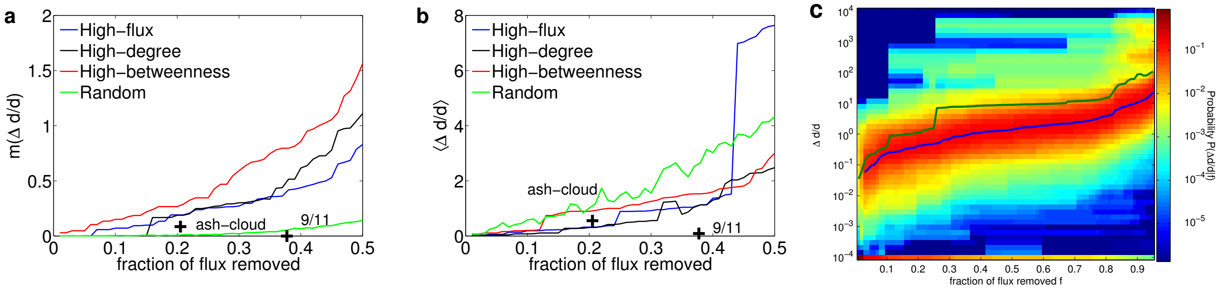

Figure S5: Inflation of effective distances. (**a**) The global impact measure $m(\Delta d/d)$ as an increasing fraction of network traffic is removed, either in order of decreasing centrality measures or randomly. We note $m(\Delta d/d)$ increases when a small number of high centrality airports are removed but remains relatively unaffected when random airports are removed. The effect of the ash cloud event (events are marked by crosses) falls between random and targeted attacks. Surprisingly, distance increases more due to the ash cloud closures than the 9/11 closures, even though the latter removes more flux. (**b**) Same as (a) but with the global impact measure $\langle \Delta d/d \rangle$. Random attacks appear stronger than high-centrality attacks due to impact heterogeneity. (**c**) Conditional distribution of normalized distancing $\Delta d/d$ between pairs of airports. As more high-centrality airports are removed from the network (here based on node flux) the distribution of distancing broadens, showing that more shortest paths become longer in the attacked network. The first peak marks the shortest paths from and to Khon Kaen, Thailand, an airport that is almost exclusively tied to Bangkok, which is removed at 25% flux reduction, leaving Khon Kaen only very weakly connected to the network. The mean (green line) reacts sensitively to the appearance of additional small peaks while the median (blue line) is more stable. There are always pairs of airports that are not distanced, and in the plot they appear binned in the smallest plotted value of $\Delta d/d$, explaining the colored stripe along the bottom of the plot. Note that the probability of zero distancing decreases as an increasing fraction of the system traffic is removed.

S4.3 Europe as a global bridge

We show in the main text that the size of global shifts due to the ash cloud closures are unexpectedly large for the amount of traffic that they remove. Furthermore, almost all of the geographically clustered events (of a scale comparable to events that occur naturally) that lead to relatively strong global shifts are located in Europe. Fig. S6 shows the global shifts caused by geographically clustered events centered at different locations, that either affect the same geographical radius as the ash cloud or close the same number of airports. It is clear that only disruptions to Europe lead to high $m(\Delta d/d)$. We can see that the large global shifts coincide with disruptions that close a set of airports with high aggregate betweenness centrality. However, we also note that there are clear discrepancies between the size of global shifts and aggregate betweenness, since airports in the east coast of the US and in Eats Asia remove medium to large amounts of aggregate betweenness but lead to negligeable global shifts. This indicates that the large aggregate betweenness centrality of airports in Europe does not fully explain why the region is so crucial to proper network function.

The network is especially vulnerable to the removal of European hubs because together they provide global connectivity that is not easily replaceable. That is, Europe is a bridge between regions that are otherwise poorly connected, making its removal especially damaging. On the other hand, it is a good substitute for many of the global connections lost when North American airspace is closed. This exceptional importance is partly captured in Table S2: We collapse each region into a super-node and compute betweenness centrality in the resulting network. Europe not only has the highest betweenness to begin with, it is also the only region to experience an increase in the 9/11 scenario, indicating that alternate routes through Europe are the best backups in the network. On the other hand, following the ash cloud, Europe decreases in betweenness-centrality and there is no single alternative: rerouting occurs through all of North America, the Middle East and East Asia.

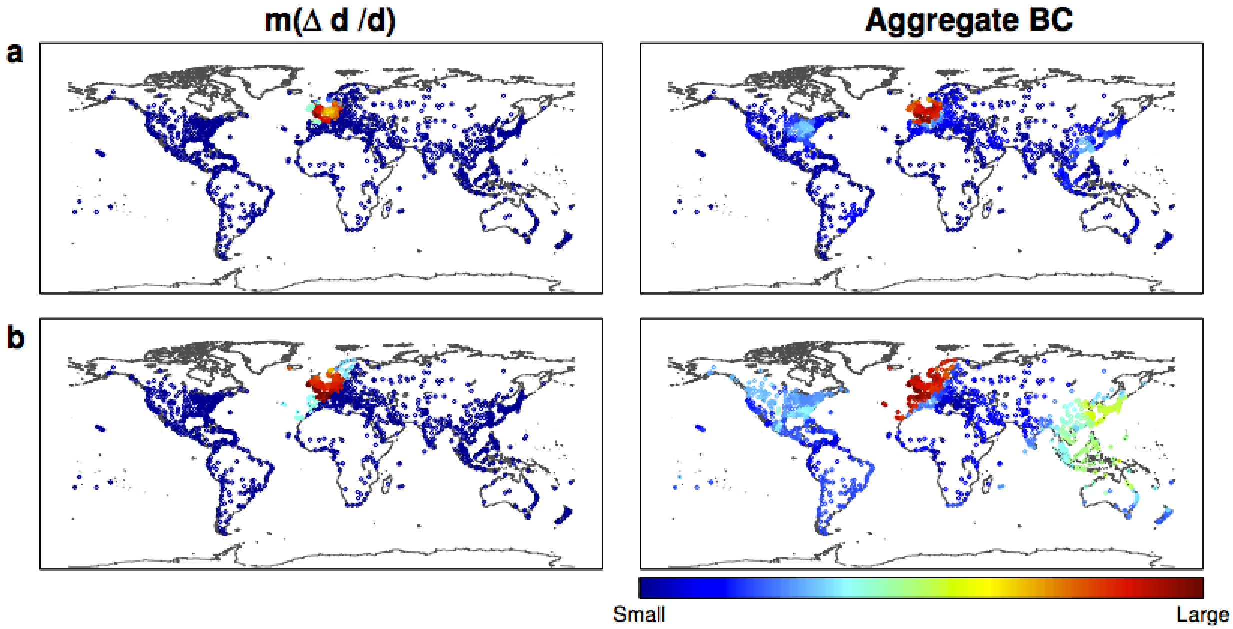

Figure S6: The global impact of geographical attacks. (**a**) At each airport we center a geographically clustered attack that removes a similar geographical radius to the ash cloud closures and compute different metrics for this attack: global shifts $m(\Delta d/d)$ and the sum of the betweenness centrality of all closed airports (aggregate BC). Global shifts are only large for disruptions clustered in Europe. The aggregate BC coincides with the strength of global shifts to some extent: European disruptions exhibit the highest shifts and aggregate BC but disruptions in North America and East Asia also remove high betweenness airports without leading to significant shifts. (**b**) Same as (a) but disruptions close the same *number* of airports as the ash cloud disruptions.

	Intact WAN	Ash-cloud	9/11
North America	0.46	0.61	0
Central America	0	0	0
South America	0	0	0
Europe	0.54	0.18	0.82
Africa	0	0	0
Middle East	0	0.071	0
Central Asia	0	0	0
East Asia	0.25	0.46	0.25
Southwest Pacific	0	0	0

Table S2: Betweenness centrality of regions. The values in the table give the normalized betweenness of the different regions in the WAN. To compute this metric each region is reduced to a regional network containing only nine nodes so that the strength of the Africa to Europe link in the reduced network, for example, is equal to the sum of the strengths of all links in the original network that join Africa and Europe. Self-links in the regional network are ignored. Europe is crucial in sustaining global interconnectivity: Europe has the greatest initial betweenness and upon its removal many regions are needed to enable appropriate substitute routes, while Europe can alone offer the needed substitute routes when North American airports close.

S5 Characterizing the distribution of airport susceptibilities

S5.1 From single events to impact ensembles

To compare the ash cloud and 9/11 closures to random and geographical events, we investigate impact over the ensembles of geographical events and of random events that remove a given amount of traffic. Similarly, we can generalize beyond specific high-centrality disruptions (these are deterministic disruptions) to an ensemble of high-centrality disruptions that remove a given amount of traffic by considering the betweenness-, flux- and degree-targeted attacks together. We use two different methods to obtain the distribution of susceptibility from these ensembles. The first method is to consider the ensemble average of the susceptibility at each airport, which yields a susceptibility distribution with one value for each airport. We refer to this distribution as a *type 1* distribution. The other approach is to consider the distribution of every susceptibility measure in the ensemble – we call this a *type 2* distribution. Both approaches yield similar scaling exponents (see Fig. S7a).

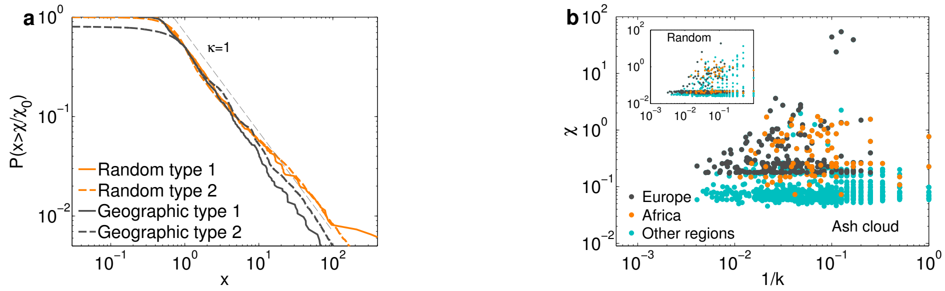

Figure S7: Impact averaged over random and geographical ensembles. (**a**) Airport susceptibilities over geographical and random ensembles exhibit the scaling $p(\chi) \sim (\chi)^{-(\kappa+1)}$ where $\kappa \approx 1$. (**b**) In the ash cloud event (as for other geographical attacks) high impact is focused on airports near the disruption epicenter. Airports in Europe and Africa are more susceptible than airports in other regions, which just exhibit a base level of susceptibility due to the removal of Heathrow. On the other hand, in the case of random disruptions (inset) airport susceptibility is uncorrelated with geography.

S5.2 The differences between geographical and random disruptions

Although the scaling exponents of geographical and random attacks show similar behavior, we can see that there are some differences between the two types of disruptions. Most importantly, in the geographical disruptions the impact tends to be stronger close to the disruption epicenter (see Fig. S7b) while in random disruptions (as in high-centrality attacks) impact is distributed similarly across all regions (see Fig. S7b inset). Because airports in a region will tend to connect through more central airports in this same region, they are highly directly susceptible to geographic disruptions. Thus, in the geographical events, the tail of the susceptibility distribution is driven by the direct susceptibility of airports in regions geopolitically close to the epicenter. In the case of random disruptions the direct susceptibility of randomly distributed airports will drive the tail.

	κ	$x_{\min}$	p-value
Geographical control A	1.0499 ± 0.0505	0.9314 ± 0.2836	0.8970
Random control A	1.0528 ± 0.0524	1.0602 ± 0.2604	0.1570
High-Centrality control A	2.0473 ± 0.3964	2.2181 ± 0.4366	0.4660

Table S3: Fitting a power-law distribution to the susceptibility distributions. The p-values > 0.05 indicate that we cannot reject the hypothesis that the data distributions were drawn from a power law. Thus, all of the disruptions are consistent with the hypothesis that airport susceptibilities are power-law distributed and all random and geographical disruptions are statistically similar, with the scaling exponent $\kappa \approx 1$, while in targeted attacks susceptibilities are more narrowly distributed with $\kappa \approx 2$. It is interesting to note that as more traffic is removed (that is, when we consider control B attacks instead of control A) the difference between high-centrality disruptions and random/geographical disruptions becomes more pronounced.

S5.3 Fitting the scaling exponent of the susceptibility distribution

The susceptibilities to random, geographical and high-centrality ensembles are plausibly power-law distributed. Using the methodology introduced in [5] we fit the empirical observations to the power-law form

$$p(x|x_{\min}, \kappa) = \frac{\kappa}{x_{\min}} \left(\frac{x}{x_{\min}} \right)^{-(\kappa+1)} .$$

To ensure the plausibility of this model for the data, we then test the null hypothesis that the empirical distribution is as close to distributions generated from the model as real power laws are to distributions generated from the model-fit to these true power laws [5]. All the p-values for this test are large enough that we cannot reasonably reject the null hypothesis and thus the power-law fit is consistent with the empirical data. We show the p-values along with the fitted parameters and their standard error, for type 1 distributions in Table S3. These results clearly support the statement that the susceptibility to central attacks is more narrowly distributed than that of random and geographical attacks. It also supports the observation that random and geographical disruptions exhibit similar scaling exponents. Additionally, the exponent values match the exponents that we derive using our theory.

S6 Hierarchical structure of shortest paths

S6.1 Modeling the structure of efficient routes

In the main text we use the hierarchical structure of the WAN to motivate the distinction between direct and indirect impact and to explain how direct impact at an airport affects its dependents indirectly. Here we lay out the evidence for this hierarchical structure and present some of the characteristics in more detail.

The shortest paths in this network are predominantly *hierarchical*, meaning that they consist of two segments (either of which can be of length zero) with a strict ordering: the first segment travels through airports in order of increasing centrality, and the second in order of decreasing centrality .Efficient navigation commonly occurs along hierarchical paths in networks with strong degree heterogeneity coupled with the tendency for increased connectivity between nodes that are close in a metric space [6]. Ranking airports according to betweenness and comparing ranking with the ordering of airports along each shortest path, we find that 88% of the paths are hierarchical. Meanwhile, ranking according to flux (degree) instead we find that 70% (68%) are exactly hierarchical. This strongly supports the assumption that network routing is hierarchical.

Using the hierarchical structure, the shortest path from airport i to airport j can be decomposed into a segment that goes from i to the most central airport on the path, call it k, and then from this airport to j. Then, the mean distance from i is given by

$$d_i = \frac{1}{N} \sum_{j,k} \sigma_{ij}(k)(d_{ik} + d_{kj}) \tag{S1}$$

where N is the total number of airports and $\sigma_{ij}(k)$ is an indicator function that is equal to one when k is the most central airport on the shortest-path from i to j. In the limit where most of the pathways from i focus onto the same central airport, call it Ω:

$$d_i = d_{i\Omega} + d_\Omega - d_{i\Omega}/N \approx d_{i\Omega} + d_\Omega \tag{S2}$$

where $d_\Omega = \langle d_{\Omega j} \rangle_j$. The term $d_{i\Omega}/N$ is a small correction for the double counting that occurs because d_Ω includes the path from Ω to i. If the same hub Ω is used by all airports in the network, Eq. S2 holds for every airport. In this case Ω must have the minimal average distance from other airports: $d_\Omega = \min(d_i)$.

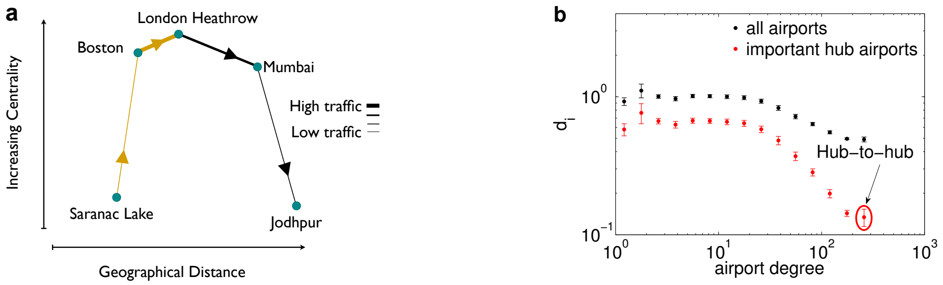

Figure S8: Effective distances and hierarchy in the WAN. (a) The structure of shortest paths with multiple stops can be decomposed into a segment of increasing centrality, towards the center of the network, and a second segment of decreasing centrality. This is a reasonable approximation for the most efficient paths in a hierarchical network such as the WAN. The effective distances are captured by the length of path segments in the cartoon. (b) The median effective distance from airport d_i and the median effective distance to the most important hubs only (airports connecting to more than 200 important destinations). We note that these distances between hubs are very small compared to all other distances, consistent with the idea of a network core.

When this model holds exactly for all airports, the shortest-path tree rooted at every airport will be the same, modulo the root. Thus, the union of all the links that are in the shortest-path tree of at least one node, which we call $\mathcal{U}_{sp}$, is just this tree. In fact, this tree will be a star, where all airports connect directly to the center and from there to every other airport.

The WAN exhibits less regularity in the shortest-path structure, and $\mathcal{U}_{sp}$ is only roughly tree-like. However, this model is a good approximation of the shortest-paths in the WAN given small modifications. In the WAN there is local structure due to spatial constraints [7] and thus airports that are geographically close will be reached without traveling through the center, and some long-range paths will include short-range connections on the way to the center rather than a direct connection to the center. Thus, most paths will be hierarchical but multi-stepped, like the example in Fig. S8a. Furthermore, the center of the network, rather than being an individual airport, is more appropriately modeled as a core set of airports that have strong connections and small distances between each other (see Fig. S8b). In the section below we show evidence that the WAN has a strong core.

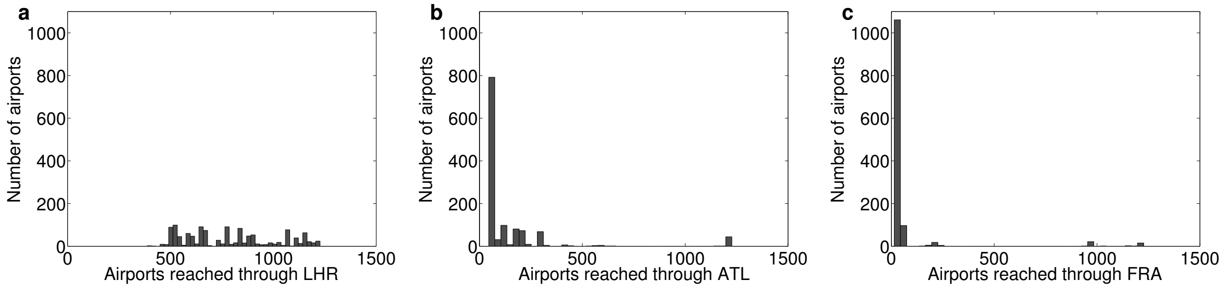

Figure S9: Heathrow's central role. (**a-c**) Histograms of the number of paths from different airports that go through Heathrow (LHR), Atlanta (ATL) and Frankfurt (FRA) respectively. Although Frankfurt and Atlanta are important hubs (they have the highest degree and flux centrality, respectively, in the WAN), Heathrow clearly provides routing for a much larger fraction of the routes from a much broader set of airports.

S6.2 The center of the network

Heathrow (LHR) is the center of the WAN according to a number of criteria. On the simplest level, it has the minimal average distance from all other airports: $d_{\mathrm{LHR}} = \min(d_i)$. It also has the highest betweenness centrality in the network (0.0155 of the total betweenness centrality of airports in the WAN is focused on Heathrow). Furthermore, we can see that most airports reach more than half of their destinations through LHR (see Fig S9).

Because LHR is so central, the shortest-path tree rooted at LHR is in fact a good representation of a shortest-path backbone for the entire network, approximating the ansatz for $\mathcal{U}_{\mathrm{sp}}$. To confirm this, we measure the average difference between Heathrow's shortest-path tree (SPT) and those of all other airports. The dissimilarity between two SPTs can be quantified using the parent-dissimilarity measure $z_p(T_i, T_j)$. The SPT of a node i in a connected network can be conveniently represented as a vector T_i such that $T_i[k]$ is the parent of airport k in the SPT rooted at i; conventionally $T_i[i]$ is taken to be zero. The parent dissimilarity is then simply the Hamming distance between tree vectors. We find that the SPT rooted at LHR has the smallest average dissimilarity from the SPTs rooted at every other airport. That is, LHR satisfies $\mathrm{argmin}\langle z_p(i, j)\rangle_j$. The z-score for Heathrow's average dissimilarity is -1.84, indicating that it is significantly small (p-value 0.0329).

Further evidence for Heathrow's role as the center of the network is how the network changes when it is removed. If shortest paths throughout the network use the same small set of hubs, the average dissimilarity in the network will be low, while a network with a more decentralized path structure has a high average dissimilarity. We find that the average dissimilarity in the WAN increases strongly when we remove LHR (43%) but does not show much change upon the removal of other individual airports, even hubs – it increases by 3.5% when Hong Kong International, the second highest betweenness airport is removed, and actually decreases by 8.0% and 3.2% when the highest flux and degree airports are removed (Atlanta and Frankfurt, respectively). This indicates that Heathrow has a strong centralizing effect on the network. In fact, the removal of Heathrow alone is enough to cause a system-wide airport response with the scaling characteristics of an attack targeting the center of the network , while removing any other single airport, even those of high centrality, will not lead to a system-wide disruption.

Although Heathrow is in many ways the most important hub in the network, it does not provide connectivity between all airports. The WAN exhibits a hierarchy of hubs, from global to regional [8]. We can think of Ω as a "fuzzy" center: a set of hubs that play an important global role rather than a single most-important hub, with Heathrow being the most prominent element of this set.

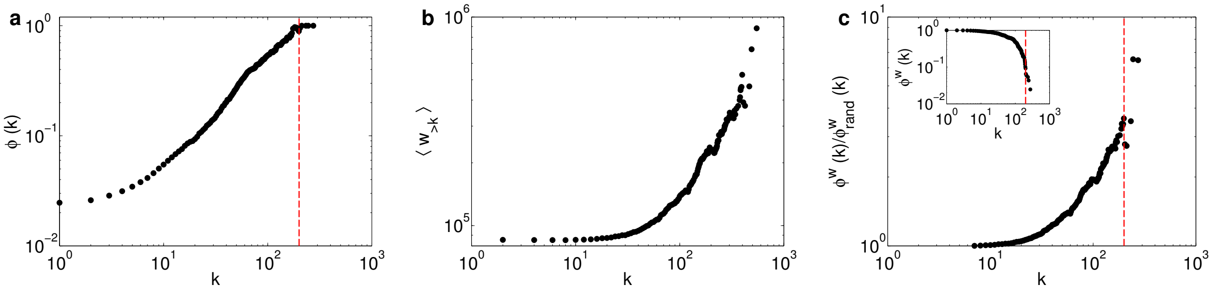

Figure S10: The core of the network. (**a**) The rich-club coefficient of the WAN shows that the highest degree airports (right of the broken red line) are completely interconnected, forming a clear core structure. (**b**) The mean strength of connections is increasingly strong as we consider connections between airports of increasing degree. (**c**) The weighted rich-club coefficient of the WAN normalized by the value of the same coefficient in a network retaining the topology of the WAN but where weights are shuffled (inset shows coefficient before normalizing). This exposes the tendency for the connections between the most central airports to be stronger than would be expected based on their degree alone, showing evidence for a core.

Can we find clear evidence that the WAN has such a core? On the simplest level, the core airports are the small set of airports that are in the tails of all the typical centrality distributions (see Fig. 2 in main text). Most of the evidence points to a core of less than 10 airports – a vanishingly small fraction of the total number of airports (see Fig. 2 or Fig. S10 for example). An approach to capture the presence of a core is through the **rich-club coefficient** [9]. The un-weighted rich-club coefficient measures the fraction of edges connecting a pair of nodes out of the maximum number of connections that would be possible [9]. Let $N_{>k}$ represent the number of nodes of degree greater than k and $E_{>k}$ be the number of edges connecting two nodes in this set.[1] The topological rich-club coefficient is given by

$$\phi(k) = \frac{2E_{>k}}{N_{>k}(N_{>k} - 1)}. \tag{S3}$$

We can see in Fig. S10a that this measure shows the presence of a tightly interconnected core.[2]

Not only are the central airports more strongly interconnected than the rest of the network, but the strength of connections is greater between these central airports. To see this, we show in Fig. S10b the average strength of connections $\langle w_{>k} \rangle$ between nodes of degree greater than k.

We can go further and investigate what fraction of the total traffic originating from a group of airports travels between this group, extending the rich-club coefficient to incorporate the strength of connections [9]:

$$\phi^w(k) = \frac{2w_{>k}}{\sum_{i|k_i>k} f_i}. \tag{S4}$$

In this case, because the hubs are important global connectors, $\phi^w(k)$ does tend to decrease with k. However, if we normalize $\phi^w(k)$ by comparing it to the same score for a network that preserves the topology of the WAN but where the weights are shuffled, we find that in the WAN the hubs share a much larger fraction of their traffic than we would randomly expect (see Fig. S10c). This is especially accentuated for the few highest degree airports, again indicating the presence of a strong core.

[1]We can use a centrality measure other than degree (flux or betweenness) but we simply note that all the results below still hold for these two measures and instead focus on degree, which plays a more prominent role throughout our analysis.

[2]Note that we have not normalized the coefficient by a null-model since for our current purposes it is important to determine the presence of a core, not if it is statistically significant in some manner.

S7 Interpreting the decomposition of susceptibility and estimating background impact

In the main text we show evidence that airport susceptibility can be decomposed into a direct and an indirect component:

$$\chi_i = \chi_{i,\text{indirect}} + \chi_{i,\text{direct}}. \tag{S5}$$

We can relate the direct susceptibility to a change in the effective distance along a direct connection from the airport and the indirect susceptibility to further removed changes in effective distance, including a global component to change. To do this we first recall that susceptibility is defined as $\chi_i = m_i(d') - m_i(d)$. Following the structure of shortest paths discussed in Sec. S6, the path of median distance $m_i(d)$ from an airport i can be decomposed into two segments (either of which may include more than one direct connection): the first from i to the core of the network, and the second from the core to an airport that is approximately at a median distance from the core. This is analogous to the decomposition of mean distance from an airpot in Eq. (S2). Thus, the change in $m_i(d)$ under a disruption, i.e. the susceptibility, can also be decomposed in this manner. In the idealized case that the path of median distance is the path between the same pair of airports before and after the disruption, we can literally interpret these as changes to the respective segments of this fixed trip. Although in the WAN changes can deviate from this idealized case, the star-like structure of the shortest-path backbone means that this is a good approximation.

We can write an expression that captures this decomposition of the average change in effective distance from the perspective of individual airports. This is most neatly done in terms of Δd_i, the change in mean distance from airport i, since this quantity can be expressed in terms of simple sums of distances. Because of the star-like structure of the shortest-path backbone, both the susceptibility and the change in mean distance from an airport follow the decomposition outlined above, although they are not equal (the mean is always slightly larger due to the broad distribution of distances, especially in the case of more central airports as found in [10]). Furthermore, empirically we find that χ_i is well approximated by Δd_i plus a constant shift. Specifically, a linear fit for χ_i in terms of Δd_i yields a linear coefficient b that is close to unity for all of the disruptions that we consider ($0.985 \le b \le 0.9994$), a constant term c close to zero ($1.175\text{e}-7 \le c \le 2.117\text{e}-5$) and a small normalized root-mean-squared error ($1.776\text{e}-6 \le \text{RMSE} \le 1.113\text{e}-4$).

Following Eq. (S2) the mean change in effective distance at an airport i is approximately given by:

$$\Delta d_i \approx d'_{i\Omega} + d'_{\Omega} - d_{i\Omega} - d_{\Omega} \approx \Delta d_{ij} + \Delta d_{j\Omega} + \Delta d_{\Omega}. \tag{S6}$$

Primes denote the situation after the disruption and Δd_{ij} is shorthand for the change in effective distance between airports i and j due to the disruption. As before, Ω denotes the core of the network and, slightly abusing notation, $\Delta d_{i\Omega}$ denotes the change in effective distance from airport i to the nearest core airport while Δd_{Ω} represents the mean change in effective distance from the core airports to the rest of the network. To go from the expression in the middle to the right, we have split $\Delta d_{i\Omega}$ into components $\Delta d_{j\Omega}$ and Δd_{ij}, where j is i's neighbor and a gateway to the rest of the world (note that j can be a different airport before and after a disruption). If i directly connects to the core of the network $\Delta d_{ij} = \Delta d_{i\Omega}$ and $\Delta d_{j\Omega} = 0$.

We can relate the different components of model (S6) to direct and indirect susceptibility: $\chi_{i,\text{indirect}} \approx \Delta d_{j\Omega} + \Delta d_{\Omega}$ and $\chi_{i,\text{direct}} \approx \Delta d_{ij}$. Furthermore, $\Delta d_{\Omega} \approx \chi_{\Omega}$ and this model suggests the constant background impact χ_0 is just χ_{Ω}, the susceptibility of the core. Using this reasoning in the main text we have approximated χ_0 by computing the average susceptibility at the most central, surviving airports in the network: $\chi_0 \approx \frac{1}{|\Omega|}\sum_{i\in\Omega}\chi_i$.

In the main text we found evidence that random disruptions $\chi_{i,\text{indirect}} \approx \chi_0$ for all airports while in

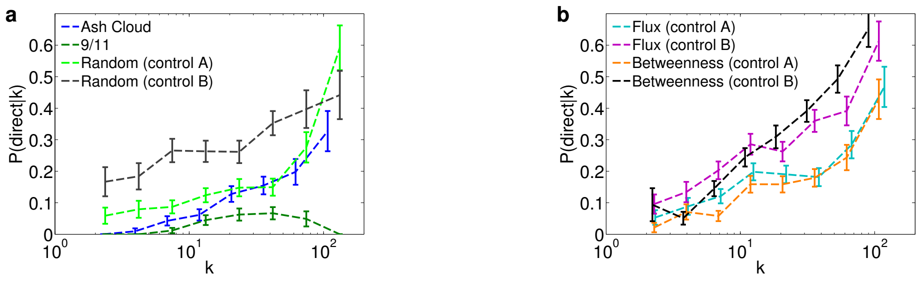

Figure S11: Variation in direct disruptions with airport degree. (**a,b**) Probability of direct impact given degree for random (a) disruptions and targeted attacks (b).

centralized attacks the indirect effect of the gateway susceptibility $\chi_{j,\text{direct}}$ plays a large role in impact scaling. Model (S6) suggests that this is because in random or geographical disruptions it is unlikely that important gateway airports j will experience crippling changes in connectivity to the core of the network so $\Delta d_{j\Omega}$ is small in the case of most airports i. On the other hand, in centralized attacks it is precisely the connections between central airports which are preferentially disrupted and $\Delta d_{j\Omega}$ is large.

S8 Scaling of susceptibility distributions when direct impact dominates

S8.1 Susceptibility and degree

In the main text we presented strong evidence for the direct impact scaling $\langle \chi_{\text{direct}} | k \rangle \sim k^{-\mu}$, where $\mu \approx 1$. The average Pearson correlation coefficient ρ over the different disruptions, including an ensemble of random disruptions, is $\rho(\ln(\chi_{\text{direct}}), \ln(k^{-1})) = 0.65 \pm 0.096$ (where errors are ± 1 s.d.), significant at a 99% significance level.

S8.2 Directly impacted airports according to disruption type

This relationship between degree and susceptibility can be used to predict the scaling exponent of $p(\chi)$ for different types of disruptions, given information about the degree distribution of directly impacted airports. We first determine which airports are directly impacted under a specific type of disruption and then approximate the functional form of their degree distribution.

Even when disruptions close airports mostly independently of their degree, such as random and geographical disruptions, the probability that an airport is directly impacted increases with degree (Fig. S11) given that central airports have a higher degree in the high-salience skeleton [11].

As can be verified in Fig. S12, a continuous approximation of the degree distribution of directly impacted airports is roughly gamma distributed:

$$p(x|\alpha, \theta) = \frac{1}{\theta^{\alpha}\Gamma(\alpha)} x^{\alpha-1} \exp^{-x/\theta} . \tag{S7}$$

To be precise, the distribution is shifted ($x = k - 2$) since directly impacted degree k has a minimum value of $k = 2$ (all directly impacted $k = 1$ airports become disconnected). We can see in Fig. S12 that α can be $\epsilon \ll 1$ smaller or larger than 1 depending on the disruption and θ is in the order of 40. The exact parameter values and other details are not important for our purposes. Rather, the general behavior of the functional

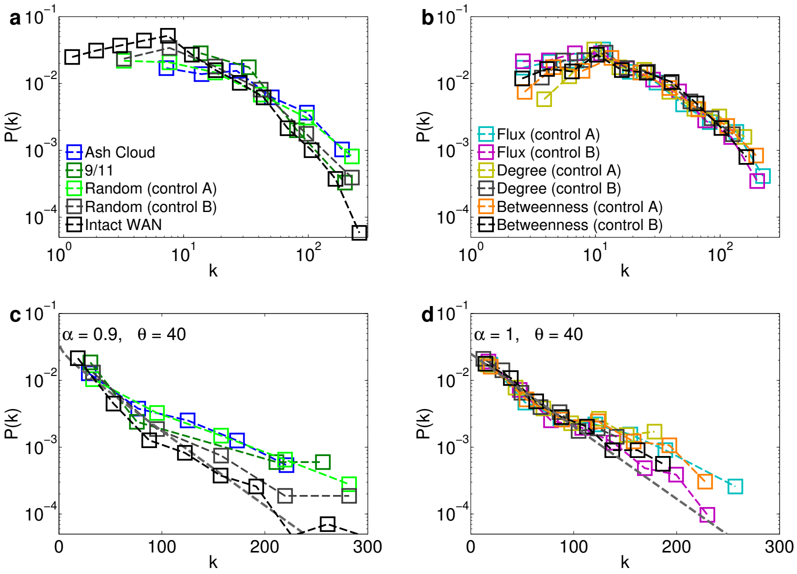

Figure S12: Degree distribution of directly impacted airports. (**a,b**) Probability density function for full degree distribution in the intact WAN and for directly impacted airports under random and real disruptions (a) and targeted attacks (b). When disruptions are centralized ϵ is slightly larger. (**b,c**) Same as (a,b) but on a log-lin axis which captures clearly the exponential decay of $p(k)$. We conclude that $p(k)$ for the directly impacted airports approximately follows a gamma distribution.

form will be useful in deriving the distribution of susceptibilities in the following section.

S8.3 Change of variables

Given the degree distribution of directly impacted airports we can accurately estimate the scaling of $p(\chi_{\text{direct}})$ using information about degree only. It is a well-known result (see [1] for example) that in general, the function for predicting y in terms of x that minimizes the mean square error is just the conditional mean of y given x, $\langle y|x\rangle$. This gives

$$\chi_{\text{direct}} = ck^{-\mu} \qquad \text{(S8)}$$

where $c \approx 10$ according to Fig. 5b in the main text. This approach is equivalent to assuming $p(\chi_{\text{direct}}|k) = \delta(ck^{-\mu} - \chi_{\text{direct}})$.

With this assumption, the probability density function of χ_{direct} can be easily derived from that of k. For convenience we drop the subscript on χ_{direct} for the following calculations.

$$\begin{aligned} p_\chi(\chi) &= \int dk\, p_k(k) p(\chi \mid k) \\ &\approx \int dk p_k(k) \delta(ck^{-\mu} - \chi) \\ &\approx \frac{1}{\mu} \int dy (y/c)^{-(1+1/\mu)} p_k((y/c)^{-1/\mu}) \delta(y - \chi) \\ &\approx \frac{1}{\mu} (\chi/c)^{-(1+1/\mu)} p_k((\chi/c)^{-1/\mu}). \end{aligned} \qquad \text{(S9)}$$

We have established above that $p(k)$ follows a gamma distribution (S7) and that according to the disrup-

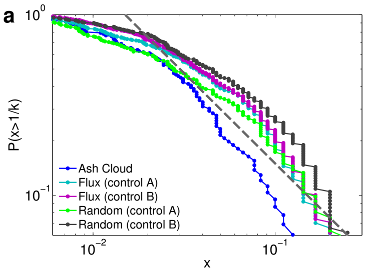

Figure S13: Direct impact distribution through degree inversion. (**a**) Cumulative distribution function of inverse degree (of directly impacted airports) for different attacks. The broken grey line marks $P(x > 1/k) = (1/k)^{-1}$, which corresponds to a scaling exponent $\kappa = 1$, in agreement with the local impact distribution of random and geographical disruptions.

tion $\alpha = 1 \pm \epsilon$ where $\epsilon \ll 1$. Approximating to first order, this yields

$$p_\chi(\chi) \approx \frac{1}{\mu\theta}(\chi/c)^{-(1+1/\mu)} \exp^{-c/(\theta\chi)} . \tag{S10}$$

Recalling $\mu \approx 1$ following the results in the main text, independent of the exact values of $\theta, c > 0$, the exponential term goes to 1 as $\chi \to \infty$, and thus

$$p_\chi(\chi) \sim \chi^{-(1+\kappa)} \quad \text{with} \quad \kappa = 1/\mu \approx 1. \tag{S11}$$

The power law scaling of the susceptibility distribution will hold for any degree distribution of directly impacted airports that varies slowly as the degree approaches small values (and therefore the direct susceptibility approaches large values). More precisely

$$p_k(k) \sim L(k), \quad \text{where} \quad \lim_{k\to 0} \frac{L(ak)}{L(k)} = 1. \tag{S12}$$

To test the result that we can recover the scaling of $p(\chi_{\text{direct}})$ from the scaling of the degree distribution, we calculate the scaling exponent of the empirical inverse degree distribution of directly impacted airports. We find that the scaling exponent $\kappa \approx 1$ as we would expect (see Fig. S13).

Thus, we have established the behavior of direct susceptibility, but our end goal is to understand the scaling of the full susceptibility distribution. Reintroducing subscripts to distinguish direct susceptibility and treating indirect impact as a constant χ_0 we have:

$$p(\chi) \sim (\chi - \chi_0)^{-2} \sim \chi^{-2} + 2\chi_0\chi^{-3} + \dots . \tag{S13}$$

If $2\chi_0 << \chi$ as it is in the real, geographical and random disruptions, the first term will dominate, and we recover the scaling of impact observed empirically.

S9 Scaling of susceptibility distributions when indirect impact dominates

S9.1 Measuring the relationship between degree and number of dependents

To explain how impact at airports that lose an important connection will disrupt the connectivity of airports that are not directly affected, we need to determine what airports heavily use the directly disrupted airports to route their traffic.

Define a dependent of i as an airport that reaches at least half of all remaining $N - 2$ airports through i.

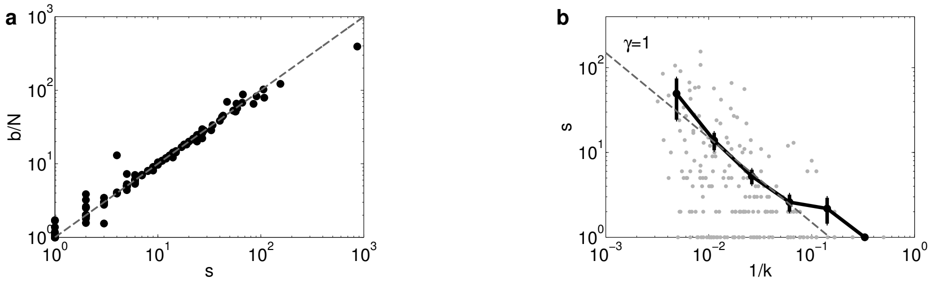

Figure S14: Scaling relationships for the number of dependents. (**a**) The number of dependents s and betweenness centrality b divided by the number of nodes N closely follow each other in the WAN. (**b**) The number of dependents of an airport scales with degree according to $s \sim k^{\gamma}$, where $\gamma \approx 1$. This is the same scaling exponent obtained for the betweenness and degree (see Fig. 6c in the main text).

To calculate the number of dependents s_i of airport i it is convenient to define the pair dependencies z_{ji} [12], which is just the number of airports that j reaches through i. Define an indicator variable s_{ji} which is one if j is a dependent of i:

$$s_{ji} = \begin{cases} 1, & \text{if } z_{ji} \geq (N-2)/2 \\ 0, & \text{otherwise.} \end{cases} \tag{S14}$$

The number of dependents of airport i is given by

$$s_i = \sum_{j \neq i} s_{ji}. \tag{S15}$$

Making the additional assumption that an airport will access all airports through the same channels, formally $s_{ji} = 1 \implies z_{ji} = N - 2$, it follows that

$$\sum_{j \neq i} z_{ji} = (N-2)s_i. \tag{S16}$$

Furthermore, the sum of the pair dependencies of airport i over all source airports j is equal to its betweenness centrality [12] (we are considering undirected betweenness given the undirected model of the WAN that we use, directed betweenness is a factor of two greater):

$$\sum_{j \neq i} z_{ji} = b_i. \tag{S17}$$

Thus the number of dependents is approximately the per-node betweenness when $N \gg 2$:

$$s_i = b_i/(N-2) \approx b_i/N. \tag{S18}$$

Because the union of shortest-path trees $\mathcal{U}_{\rm sp}$ is tree-like and in fact well-approximated by a star-like structure as described above, we find that $s \approx b/N$ as shown in Fig. S14a. This relationship between the number of dependents s and and betweenness allows us to use the betweenness centrality of an airport to estimate how many airports it affects indirectly. Specifically, we use the scaling of betweenness with degree, which exhibits the same behavior as the scaling of s with degree (see Fig. S14b), as we would expect from the argument above.

S9.2 Indirect impact narrows the distribution of susceptibility

When the most central airports in the network are directly targeted, the spread of impact from the directly impacted gateway airports to their dependents will narrow the susceptibility distribution. For the calculations that follow we make the assumption that an airport will only have one remaining gateway that experiences direct impact. This is not necessarily the case but is a reasonable approximation in the sense that in a hierarchical structure (which we show to be a good approximation in this network in Sec. S6), the least-central affected gateway airports will dominate the indirect susceptibility.

The probability that a randomly selected airport will be a dependent of a directly affected airport with s dependents is

$$sp(s) = \sum_k sp(s|k)p(k) \approx \int dk sp(s|k)p(k). \tag{S19}$$

Ignoring fluctuations, we use $s \sim k^\gamma$ (in Fig. 6c in the main text we have confirmed $\gamma \approx 1$) to make the approximation $p(s|k) \approx c\delta(k^\gamma - s)$. This yields

$$sp(s) \sim k^\gamma p(k). \tag{S20}$$

The direct susceptibility at an airport will typically affect its dependents indirectly and therefore, using similar reasoning as in Eq. (S9) we can find the scaling behavior of the distribution of indirect susceptibility:

$$\begin{aligned} p_\chi(\chi) &\sim \int dk\, k^\gamma p_k(k) p(\chi \mid k) \\ &\sim \chi^{-(1+(1+\gamma)/\mu)} p_k(\chi^{-1/\mu}) \\ &\sim \chi^{-(1+\frac{1+\gamma}{\mu})} \end{aligned} \tag{S21}$$

where $(1+\gamma)/\mu \approx 2$. Indirect susceptibility dominates when the central airports are targeted and hence the scaling above describes the full susceptibility distribution. Thus, our derived exponent $\kappa = 2$ matches the observed scaling exponent of targeted disruptions.

Deriving the scaling above in terms of the betweenness b without introducing the degree k would be more direct. However, this approach does not work because betweenness does not contain information about the direct susceptibility of the majority of airports. Most of the airports in the network (80.3%) have a betweenness centrality of zero and yet these less central airports play an important role in the direct susceptibility distribution. Thus, although betweenness helps to capture the indirect effects of central airports, degree is needed to capture direct effects at less central airports.

References

1. Rice J (2007) Mathematical Statistics And Data Analysis. Duxbury Advanced Series. Thomson/Brooks/Cole.

2. (2011) World crisis analysis whitepaper. Technical report, OAG Market Intelligence.

3. Newman M (2010) Networks: An Introduction. New York, NY, USA: Oxford University Press, Inc.

4. (2010). Air travel disruption after the 2010 eyjafjallajökull eruption. `http://en.wikipedia.org/wiki/Air_travel_disruption_after_the_2010_Eyjafjallaj%C3%B6kull_eruption`. Accessed: 20/04/2010.

5. A Clauset CS, Newman M (2009) Power-law distributions in empirical data. SIAM Review 51: 661-703.

6. Boguna M, Krioukov D, Claffy K (2009) Navigability of complex networks. Nat Phys 5: 74–80.

7. Barthélemy M (2011) Spatial networks. Physics Reports 499: 1 - 101.

8. Guimerà R, Mossa S, Turtschi A, Amaral L (2005) The worldwide air transportation network: Anomalous centrality, community structure, and cities' global roles. Proc Natl Acad Sci USA 102: 7794–7799.

9. Colizza V, Flammini A, Serrano M, Vespignani A (2006) Detecting rich-club ordering in complex networks. Nat Phys 2: 110–115.

10. Woolley-Meza O, Thiemann C, Grady D, Lee J, Seebens H, et al. (2011) Complexity in human transportation networks: a comparative analysis of worldwide air transportation and global cargo-ship movements. EPJ B 84: 589–600.

11. Grady D, Thiemann C, Brockmann D (2012) Robust classification of salient links in complex networks. Nat Commun 3: 864.

12. Freeman L (1980) The gatekeeper, pair-dependency and structural centrality. Quality & Quantity 14: 585–592.
